# Study of Microstructural, Nutritional, and Biochemical Changes in Hulled and Hulless Barley during Storage Using X-ray and Infrared Techniques

**DOI:** 10.3390/foods12213935

**Published:** 2023-10-27

**Authors:** Navnath S. Indore, Digvir S. Jayas, Chithra Karunakaran, Jarvis Stobbs, Viorica F. Bondici, Miranda Vu, Kaiyang Tu, Omar Marinos

**Affiliations:** 1 Biosystems Engineering, University of Manitoba, Winnipeg, MB R3T 5V6, Canada; indoren@myumanitoba.ca (N.S.I.); chithra.karunakaran@lightsource.ca (C.K.); 2 President’s Office, A762 University Hall, University of Lethbridge, Lethbridge, AB T1K 3M4, Canada; 3 Canadian Light Source Inc., Saskatoon, SK S7N 2V3, Canada; jarvis.Stobbs@lightsource.ca (J.S.); ibi.bondici@lightsource.ca (V.F.B.); miranda.vu@lightsource.ca (M.V.); kaiyang.tu@lightsource.ca (K.T.); omar.marinos@lightsource.ca (O.M.)

**Keywords:** synchrotron microcomputed tomography, X-ray fluorescence, mid-IR, storage of barley, microstructure, nutrition

## Abstract

Four varieties of barley (Esma, AC Metacalf, Tradition, and AB Cattlelac), representing four Canadian barley classes, were stored at 17% moisture content (mc) for 8 week. Stored barely was characterized using synchrotron X-ray phase contrast microcomputed tomography, synchrotron X-ray fluorescence imaging, and mid-infrared spectroscopy at the Canadian Light Source, Saskatoon. The deterioration was observed in all the selected varieties of barley at the end of 8 week of storage. Changes due to spoilage over time were observed in the grain microstructure and its nutrient distribution and composition. This study underscores the critical importance of the initial condition of barley grain microstructure in determining its storage life, particularly under unfavorable conditions. The hulled barley varieties showed more deterioration in microstructure than the hulless varieties of barley, where a direct correlation between microstructural changes and alterations in nutritional content was found. All selected barley classes showed changes in the distribution of nutrients (Ca, Fe, K, Mn, Cu, and Zn), but the two-row AC Metcalf variety exhibited more substantial variations in their nutrient distribution (Zn and Mn) than the other three varieties during storage. The two-row class barley varieties showed more changes in biochemical components (protein, lipids, and carbohydrates) than the six-row class varieties.

## 1. Introduction

Barley (*Hordeum vulgare* L.) holds significance as a vital cereal crop in Canada and globally. It is cultivated for purposes such as animal feed, malt products, and human consumption. Globally, 70% of barley production is allocated for animal feed, with the remainder being utilized for human consumption, including malt and food products [1]. Canada ranks as the fifth-largest producer globally, producing 9.8 million metric tonne (Mt) of barley from an area of 2.8 million hectares in 2022 [2]. Canadian barley can be categorized into two types: hulled (intact husk) and hulless (loosely attached husk), further subdivided into six-row or two-row classes [3]. The malt industry is one of the main consumers of barley in Canada and around the world. The barley intended for malt production, a prerequisite of 95% varietal purity, is important [4], and Canada excels in producing high-quality barley for the malt export market. Key quality parameters for barley consist of better germination rate, good protein content, and kernel uniformity. Superior-quality barley has less than 5% broken kernels, a protein content ranging from 9 to 12%, and a germination rate of 95% or higher [5,6].

Post-harvest storage of barley, like other cereals, is essential prior to consumption. Losses can occur during grain storage, with the extent of losses spanning from 1 to 50%, depending upon the storage conditions and characteristics [4,5,6]. The storage life of barley is influenced by both biotic factors (insects and fungi) and abiotic factors (temperature, humidity, and moisture). Among these, maintaining an appropriate moisture level is paramount. Barley is typically harvested at elevated moisture levels to mitigate harvesting losses. Therefore, drying is required to achieve a storage moisture content of 13% (wb). Elevated moisture levels during barley storage foster mold growth, culminating in post-harvest losses including diminished germination, nutrient degradation, weight loss, and toxin contamination [7]. A multitude of studies have been conducted to assess and mitigate quality and post-harvest losses in barley storage, focusing on the interaction of abiotic and biotic factors [8,9,10,11,12,13,14]. The average nutritive loss of 2.82% was found in barely stored for 12 months, protein losses were about 2.5%, and losses in fats were minimal [7]. The germination of 14% and above moisture content malting barley was found to be reduced below 95% when stored for five months [15], and high temperature and moisture can reduce barley germination to 0% at the end of nine months [13]. Significant changes can occur in free fatty acid values during the storage of hulless and hulled barley [13]. The most common fungi that affect the quality of malting barley in storage are *Aspergillus*, *Penicillium*, and *Fusarium* [16].

All agricultural food grain seeds possess unique attributes such as physical (size, shape, and colour) and composition (nutrients), which serve as distinguishing factors among them [17]. Even within the same category of cereal grains, variations in seed characteristics exist. For instance, Canadian wheat is categorized into sixteen classes, while barley is grouped into eight classes based on seed attributes. These attributes can play a role in safe storage of cereal grains. The varieties of the same grain type or class can exhibit varying degrees of resistance to deterioration within their respective habitats, as evidenced by instances where certain varieties have outperformed others during storage [18]. The inherent structure and properties of the seed might contribute to its defense mechanisms against spoilage under unfavorable conditions. In pursuit of insights, numerous investigations have been conducted to uncover such information concerning seeds, utilizing advanced imaging techniques such as X-ray and hyperspectral imaging [14,19,20,21,22,23]. These techniques have enabled researchers to extract microstructural details of seeds, composition, structural arrangements, and dynamic processes inside the plants, fruits, and seeds [24]. The utilization of synchrotron imaging has gained prominence in agriculture and food science, primarily due to limitations (experiment time, resolution, and non-coherent source) associated with conventional imaging methods (X-ray, microscopic, and digital imaging). Comprehensive applications of synchrotron X-rays have been compiled, and details can be found in the literature [15,17,25]. The application of synchrotron X-ray microtomography has been extensively used in food industries for product development and for mapping changes in the structure of food, like bread; noodle dough bubbles; the cracking of chocolate; microstructure changes due to gas exchange in products; ice cream stability under varying conditions; and the air pathways in pome fruits and in fish [17]. Synchrotron phase contrast imaging has an edge over normal absorption imaging because it can work for low-density materials like the soft tissues of plants and the differentiation of dynamic processes in tissues. Phase contrast is created by varying the propagation distance between a sample and its detector; therefore, this unique property can be utilized in the differentiation of seed features like changes in deterioration, moisture, and air volume, which have fewer density gradients. More detailed theories and their properties can be found elsewhere [26].

In a similar vein, the exploration of post-harvest dynamics in malting barley presents a promising avenue for synchrotron imaging. The quest for superior quality malting barley necessitates the presence of robust grain structures coupled with elevated germination potential and vigor [15]. Hence, a hypothesis emerges wherein synchrotron X-ray and mid-infrared technologies might be harnessed to map structural, nutritional, and biochemical changes that occur in barley varieties due to spoilage during post-harvest storage.

## 2. Materials and Methods

In this study, commercially available and certified seeds of four distinct barley classes were acquired from a private enterprise (SeCan, Niverville, MB, Canada). Two barley varieties, namely Esma and AC Metacalf, were selected to represent the western Canadian malting two-row hulled variety (2R) and the western Canadian malting two-row hulless variety (2Rhl), respectively. Additionally, two other varieties, Tradition and AB Cattlelac, were selected to represent the western Canadian malting six-row hulled variety (6R) and the western Canadian six-row hulless variety (6Rhl), respectively. Samples of each variety, about 500 g, were conditioned to achieve a moisture content of 17% (wb) using distilled water. Subsequently, these samples were stored within sealed glass jars, each possessing a volume of 1 L under ambient conditions where temperatures ranged between 22 and 25 °C. This process of conditioning and storage was recurrently executed every week for a duration spanning 8 week. The moisture content of the samples was evaluated post storage through an oven-drying method. Specifically, 10 g samples were subjected to drying at 130 °C for 19 h, and the resulting moisture content was determined [27].

### 2.1. Synchrotron X-ray Phase Contrast Microcomputed Tomography (SR-µCT)

**Figure 1:**
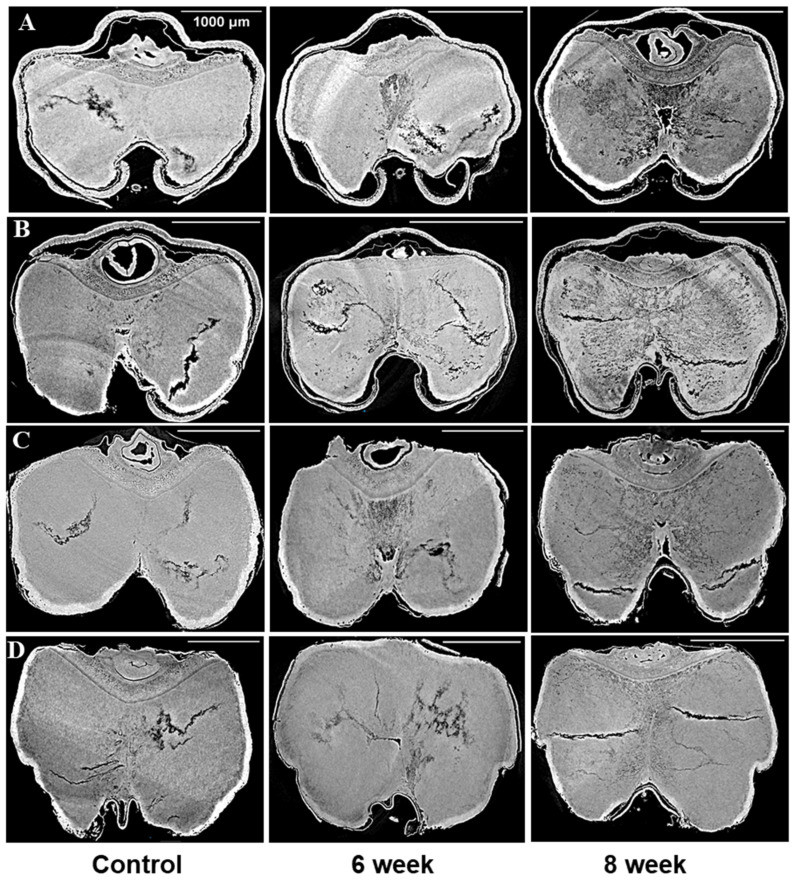
Cross sections from images of the control and 17% moisture content for four barley varieties stored for 6 and 8 weeks: (**A**) (6R) Tradition, (**B**) (2R) AC Metacalf, (**C**) (6Rhl) AB Cattlelac, (**D**) (2Rhl) Esma. The white bar in the X-ray projections represent the scalebar of 1000 µm.

**Figure 2:**
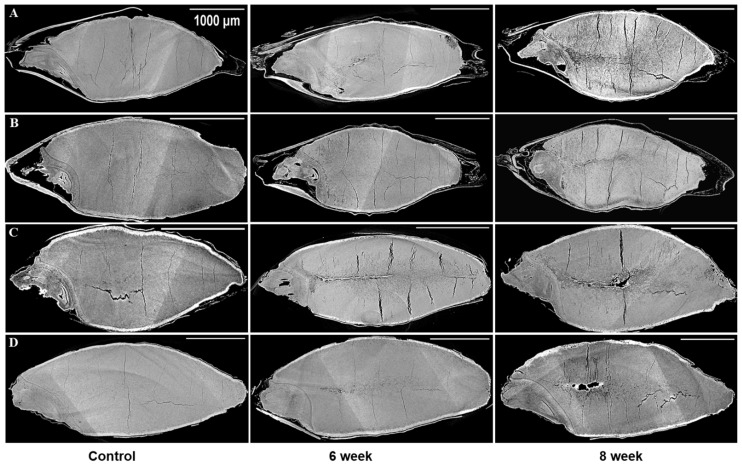
Longitudinal sections from 3D images of the control and 17% moisture content for four barley varieties stored for 6 and 8 weeks: (**A**) (6R) Tradition, (**B**) (2R) AC Metacalf, (**C**) (6Rhl) AB Cattlelac, (**D**) (2Rhl) Esma. The white bar in the X-ray projections represent the scalebar of 1000 µm.

The sample preparation, data acquisition, and data processing were carried out as per the procedures described in [28] and briefly summarized here. The imaging of the selected barley samples was completed at the BMIT-BM beamline at the Canadian Light Source (CLS) in Saskatoon, Canada. For energy filtration, two filters, namely aluminum (Al) with a thickness of 0.800 mm and molybdenum (Md) with a thickness of 0.076 mm, were used. These filters effectively modified the incident X-ray beam into a filtered white beam with an energy level approximating 20 keV. The transmitted X-rays were converted into visible images using a scintillator (LuAg 500) in combination with a detector (PCO Edge 5.0, AA-40). The acquisitions were carried out at a resolution of 3.6 µm. Fifteen to twenty individual grain kernels were randomly chosen for each variety from the glass jars. These kernels were then placed inside a plastic tube and positioned onto a sample stage, secured with reusable adhesive. The orientation of the sample was carefully adjusted to ensure its eccentric alignment on the stage, guaranteeing its sustained presence within the beam’s field of view. Subsequently, the sample was mounted onto a rotating stage positioned between the X-ray detector and the X-ray beam. The rotating stage facilitated a controlled alteration in the sample’s orientation, accomplished through increments of 0.06°, relative to the incident beam. A vital parameter in phase contrast imaging, the distance between the sample and the detector, was optimized at 5 cm to enhance contrast through the sample. Around 3000 projection images were captured for 180° rotation of the sample. Before and following the acquisition of the CT images, reference images of flat and dark signals were captured. The average of these flat and dark images was used for normalization [24,29]. The extraction of phase information was achieved through Paganin’s method, performed from a singular phase-contrast image corresponding to each projection angle. This phase retrieval process was facilitated using the UFO-kit [30]. The results of the reconstrued data are presented in Figure 1 and Figure 2, where projections from the control, 6-week, and 8-week stored barley samples are shown. The principles and theory of X-ray phase contrast imaging are available elsewhere [26,31,32].

**Figure 3:**
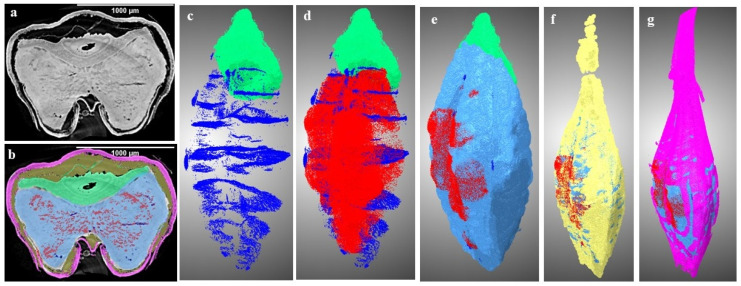
Three-dimensional visualization of segmented components of two-row hulled barley (2R: AC Metacalf) stored for 8 week: (**a**) cross section, (**b**) labelled slice, (**c**) germ (green) and cracks (dark blue), (**d**) c + changes due to deterioration (red), (**e**) d + endosperm (light blue), (**f**) e + husk gap (yellow), and (**g**) f + husk with seed coat (pink). (*n* = 1).

**Figure 4:**
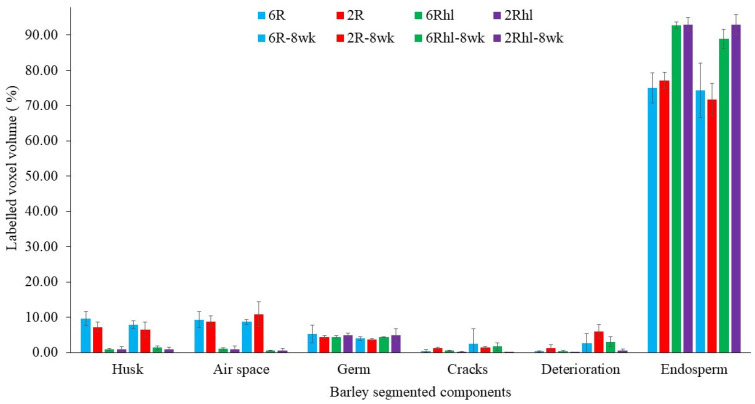
Measured labelled volume of control and 8-week stored barely varieties: (2Rhl) Esma, (2R) AC Metacalf, (6Rhl) AB Cattlelac, and (6R) Tradition). (*n* = 3).

**Figure 5:**
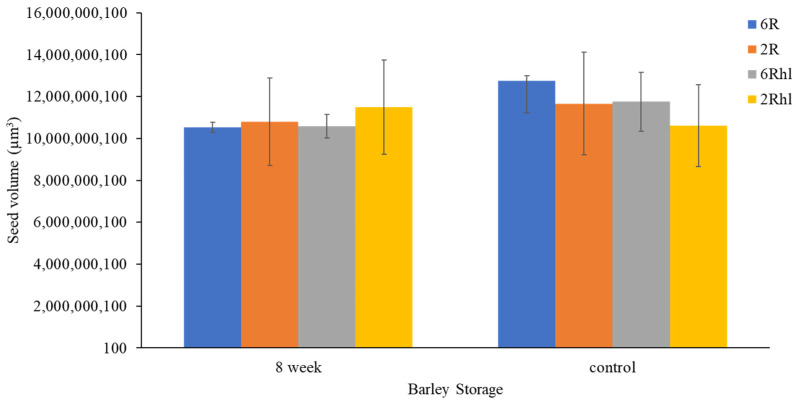
Measured seed volume of control and 8-week stored barely varieties: (2Rhl) Esma, (2R) AC Metacalf, (6Rhl) AB Cattlelac, and (6R) Tradition). (*n* = 10).

**Figure 6:**
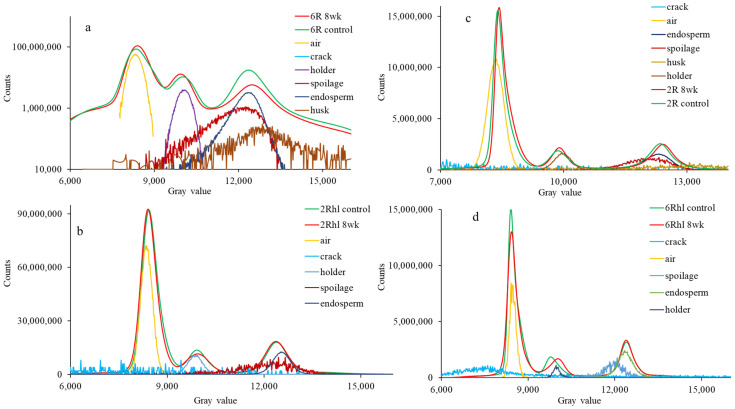
Normalized histogram of control and 8-week stored barely varieties: (**a**) (6R) Tradition, (**b**) (2Rhl) Esma, (**c**) (2R) AC Metacalf, and (**d**) (6Rhl) AB Cattlelac. Deconvoluted data are shown below the peaks in the logplot of (**a**) the 6R barley histogram.

The reconstructed data were effectively assembled in a vertical manner using the “ez_helper” module, an integral component of the UFO kit. Subsequently, a further 4 × 4 binning process was applied to ensure that the data size remained below 4 gigabytes. This operation was performed using the software “Fiji” (version 2.9.0), setting the stage for subsequent image processing. To evaluate the properties of the seed, specifically the volume and air space, an analysis was conducted on ten individual kernels per sample using a label analysis module in ORS Dragonfly software (Version: 2021.3, Object Research Systems, Montreal, QC, Canada) for both the control and spoiled sample data. Further segmentation procedures were executed to extract more intricate features from the images, including but not limited to cracks, air gaps (between the husk and endosperm/seed coat), seed coat/husk, endosperm, change due to deterioration, and germ. The initial step encompassed the creation of distinct regions of interest (ROIs) for each component of interest, accomplished through the segmentation editor in software. This enabled the segmentation of components like the germ, air spaces, and microstructural alterations indicative of spoilage. These analyses were performed to check the effect of storage and spoilage on seed microstructure; therefore, it was performed on control and 8-week stored barley samples. Visualization of these segmented components and measurement for both the control and 8-week stored samples is presented in Figure 3 and Figure 4, subsequently utilized for a comprehensive characterization of the changes in barley due to spoilage. The results of this analysis are depicted in Figure 5. Additionally, the normalized histograms of both control samples and stored samples are plotted and displayed in Figure 6. These histograms were prepared by processing 4096 bins with 0 to 65,536 image stacks.

### 2.2. Synchrotron X-ray Fluorescence Imaging (SR-XFI)

The sample preparation, data acquisition, and data processing were carried out as per the procedures described in [33] and briefly summarized here. To spatially visualize the distribution of nutrients within barley seeds, this method was used. Randomly selected barley seeds were embedded in a Leica Cryogel using liquid nitrogen. Subsequently, thin sections of these seeds were obtained with a thickness of 80 µm, employing the established procedure [34]. The thin sectioning procedure was executed using a Leica CM1950 cryostat microtome (Leica Biosystems Inc., Richmond Hill, ON, Canada). The utilization of thin sections is instrumental in minimizing distortions that may arise in nutrient maps due to the penetrative characteristics of X-rays [35]. Multiple thin-sectioned samples were positioned on Kapton tape and arranged on the sample holder. Before acquisition, microscopic images were captured utilizing a digital microscope (Motic DM143, Motic, Kowloon, Hong Kong). Following this preliminary step, the SR-XFI imaging of the samples was performed. Notably, one sample representative of each specific barley class was subjected to SR-XFI analysis to construct nutrient distribution maps of barley sections.

**Figure 7:**
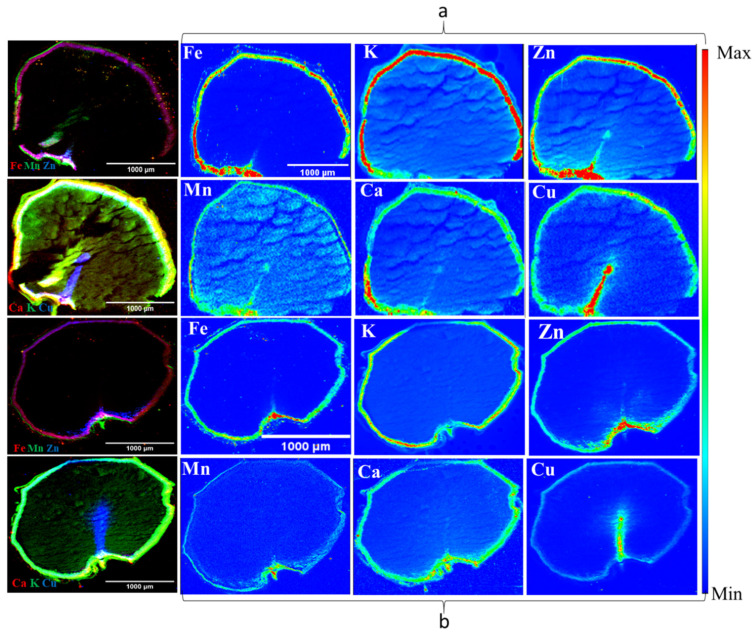
X-ray fluorescence images of six-row AB Cattlelac barely variety, control ((**a**) **top**) and 8-week stored samples ((**b**) **bottom**), showing nutrient distributions (**left**: nutrient in group distribution and **right**: individual distributions) (*n* = 1).

The data were collected at the BioXAS-Imaging beamline at the CLS, Saskatoon. The in-vacuum undulator of the beamline provides a high spectral brightness source for X-rays. In this study, the incident beam energy was set to 15 keV. The Kirkpatrick-Baez (K-B) micro-focusing mirrors were used to micro-focus the monochromatic X-ray beam spot size to 5 µm × 5 µm. Data were collected in continuous bi-directional fly-scanning mode with a step size of 5 µm and 100 ms dwell time. The Vortex-ME3 silicon drift X-ray detector (Hitachi High Technologies Science American, Inc., Chatsworth, CA, USA) was at 45°, and the samples were in a 90° stage configuration to the incident beam. The detector to the sample distance was set at 3.5 cm, and each seed section took about 6–8 h to scan. Data were acquired using PyAcq (CLS, Saskatoon), an in-house developed multi-threaded python-based software. Calibration of the nutrient peaks and data fitting was carried out using PyMca software (5.6.7) [36], and the results are presented in Figure 7.

### 2.3. Mid Infrared (Mid-IR) Spectroscopy

The sample preparation, data acquisition, and data processing were carried out as per the procedures described in [33] and briefly summarized here. To quantify the biochemical constituents encompassing the proteins, lipids, and carbohydrates, mid-infrared (IR) spectroscopy was carried out [37]. For the comprehensive analysis of barley biochemistry on a larger scale, sample sets consisting of 25 to 30 grain kernels were chosen randomly from the storage jars. These selected samples were then subjected to cryogenic grinding to produce finely powdered material. Subsequently, these fine flours, amounting to 4.85 mg each, were combined with 385 mg of potassium bromide (KBr), resulting in the creation of three distinct technical replicates for each sample. The mixture of fine flour and KBr underwent further grinding using the cryo grinder, ensuring homogeneity. From this homogenized mixture, equal portions were extracted, and these portions were subsequently subjected to the pressing process. A hydraulic press (Auto-CrushIR, PIKE Technologies, Madison, WI, USA) was employed for this purpose. Three different pressures were applied during the pressing procedure, specifically 53.93, 34.32, and 14.70 kPa. Each pressure was maintained for 3 s. This sequence of pressing yielded the formation of three individual pellets for each sample. These pellets were standardized in size, measuring 13 mm in diameter.

**Figure 8:**
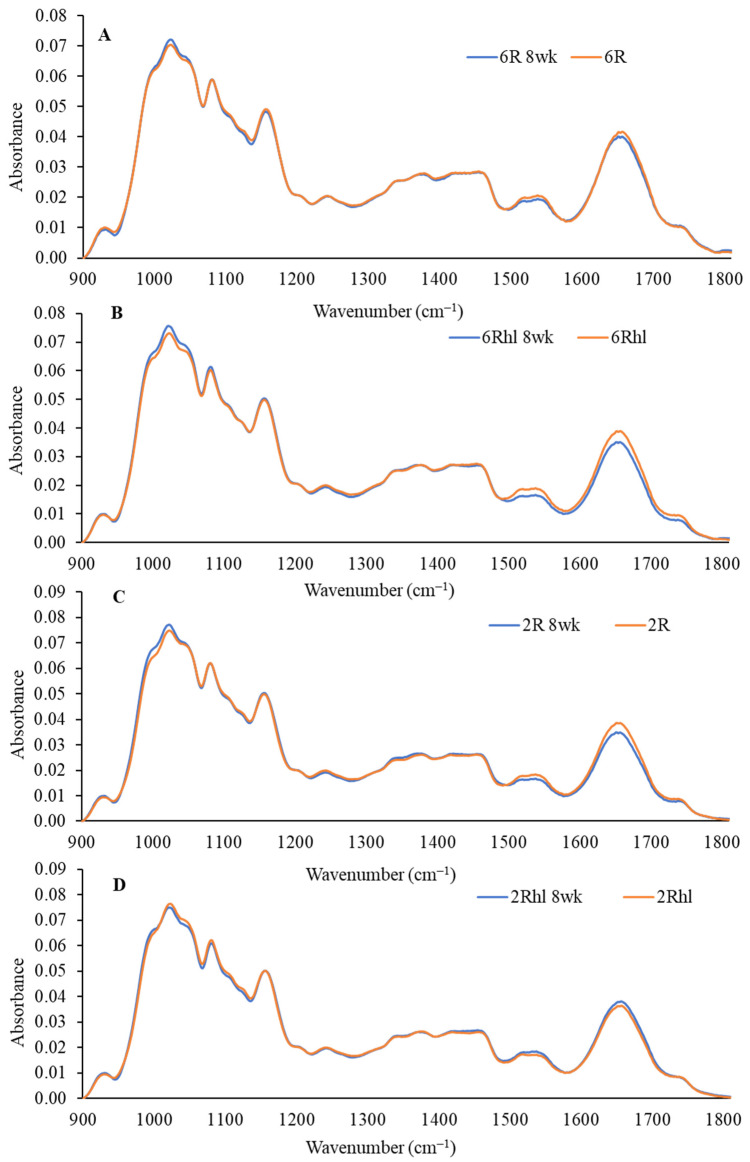
Normalized mid-IR spectra of three sample pellets of four barley classes ((**A**) 6R, (**B**) 6Rhl, (**C**) 2R, and (**D**) 2Rhl) of control and 8-week stored samples (*n* = 3).

**Figure 9:**
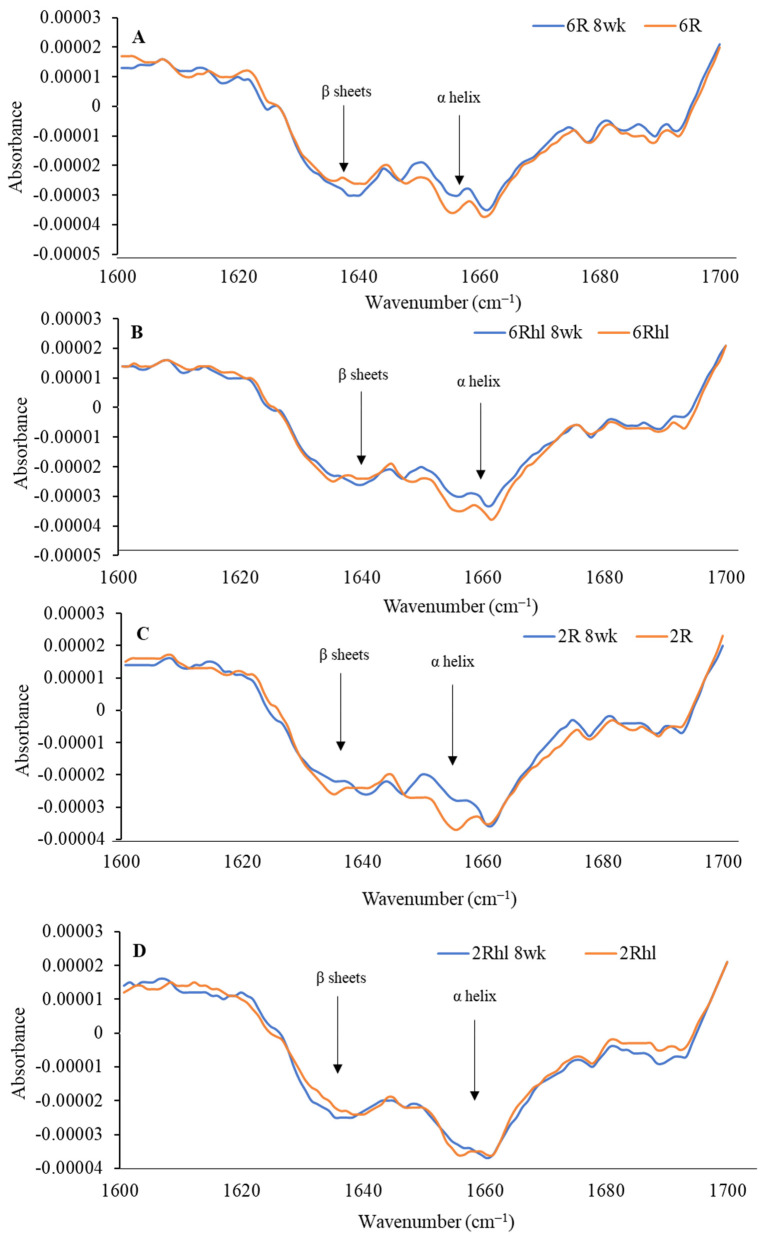
Second derivative spectra of control and 8-week stored barley classes ((**A**) 6R, (**B**) 6Rhl, (**C**) 2R, and (**D**) 2Rhl) for analysis of changes in protein structure.

**Figure 10:**
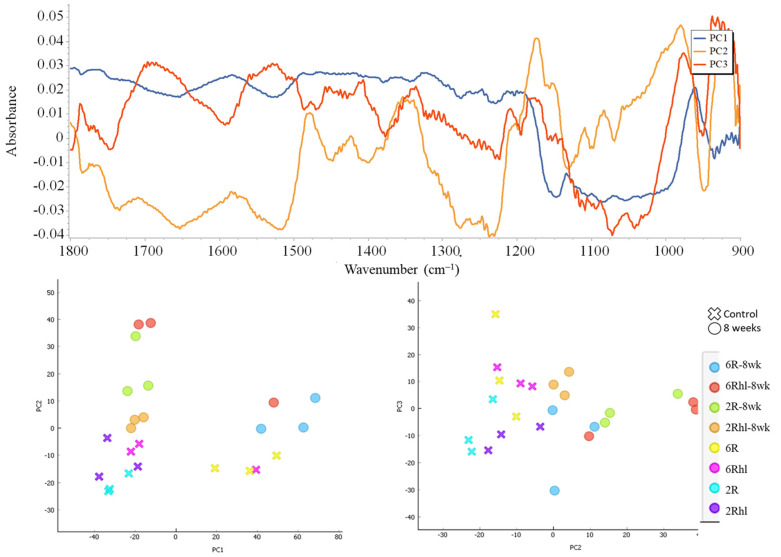
(**Top**): PC data loading plot and (**bottom**): PC score plots of control and 8-week stored samples of four barley varieties.

The samples, now in pellet form following the previously outlined process, were positioned within a sample wheel designed for data collection. For the data acquisition, a FTIR microscope (Cary 670, Agilent Technologies Inc., Santa Clara, CA, USA) was employed. This microscope was equipped with a bulk analysis accessory and a Deuterated Lanthanum α-Alanine-doped TriGlycine Sulphate (DLaTGS) detector, which was maintained at a low temperature through thermoelectric cooling. The spectral data were collected within the range of 4000 to 900 cm^−1^, at a resolution of 4 cm^−1^. A total of 64 scans were co-added to ensure reliable and accurate data. After data acquisition, the processing and analysis steps were executed using the Quasar software (version 1.5.0) [38]. The resulting spectra were visualized and presented by plotting them in Spectragryph (version 1.2.16.1). A Principal Component Analysis (PCA) was also performed using Quasar software, and all the results of the mid-IR analysis are presented in Figure 8, Figure 9 and Figure 10. This analytical approach harnessed the capabilities of mid-IR spectroscopy to deduce the content of proteins, lipids, and carbohydrates within the pelleted samples, thus contributing to a comprehensive understanding of the barley’s biochemical composition during 8-week storage.

### 2.4. Statistical Analysis

The paired *t*-test was performed on the results of 3D segmentation analysis. It was performed for each pair (control and 8 wk storage) of barley class groups to check the significant differences for components (husk, germ, volume, air space, change due to spoilage, endosperm, and cracks).

## 3. Results and Discussion

Upon visual inspection, it was evident that the stored samples of all the selected barley varieties exhibited varying degrees of deterioration by the conclusion of the 8-week storage period. This deterioration was observed after visual inspection of the stored barley samples. Fungal infection was detected in the germ area of the stored barley varieties. This observation underscores the adverse impact of storage conditions on the barley, resulting in the growth of fungi. Additionally, the deterioration process was accompanied by the discoloration of the husk in the spoiled samples. The presence of fungal infection and the discoloration of the husk serve as tangible indicators of the spoilage process and the subsequent alteration in the overall quality and condition of the stored barley.

### 3.1. Microstructural Changes

The microstructures of all the chosen barley varieties were subjected to analysis by utilizing the SR-µCT projections that had been gathered. In doing so, the structural features of the seeds were closely examined, revealing notable disparities between the control samples and stored barley samples. In Figure 1 and Figure 2, these distinctions are visually depicted. The visual analysis of the X-ray images of control samples unveiled the presence of pre-existing cracks and air gaps within the seed endosperm. Interestingly, these cracks initiated from the crease region of the seed and subsequently propagated throughout the endosperm. This structural attribute was particularly noticeable in the selected barley samples. The shape of the inspected seeds from the same variety was observed to remain relatively consistent during the storage period, as illustrated in Figure 1 and Figure 2. However, beneath this externally unaltered appearance, significant internal microstructural transformations occurred with storage.

A novel revelation of this study was the identification of air gaps between the endosperm and the husk, which adheres closely to the seed coat in hulled barley varieties. These air spaces contribute to the overall air space of the kernel, including the presence of cracks and pores. Deterioration within the spoiled samples was characterized by shifts in grey values and better contrast with edge enhancement due to SR-µCT. The boundaries of the seed microstructure became more apparent due to the enhanced contrast achieved through phase imaging, as exhibited in Figure 1 and Figure 2. This capability to visualize structural details is crucial for understanding the mechanisms of spoilage related changes within the seed microstructure. The discernible features observed in both cross sections were subjected to further processing and segmentation, leading to specific measurements aimed at evaluating the extent of spoilage among the different classes of barley (Figure 3). Notably, in both the six-row and two-row hulled varieties, the presence of air space beneath the husk was detected. This structural feature may contribute to spoilage, particularly at unsafe moisture conditions. The changes attributed to deterioration were more pronounced in the hulled varieties belonging to both the six-row and two-row types of barley. However, among the selected barley varieties, both the six-row varieties, the hulled variety (6R) Tradition and the hulless variety (6Rhl) AB Cattlelac, showed higher deterioration than the two-row varieties.

The measured voxel volumes of segmented components were estimated from the total labelled seed volume voxels, as shown in Figure 4. The two-row AC Metacalf variety emerged as the most affected among the four selected barley varieties, with an estimated change due to deterioration of 8% of total seed volume at the end of 8 weeks of storage (Figure 4). On the other hand, the two-row hulless variety (2Rhl) Esma demonstrated better performance, with only a 2% change due to deterioration over the same duration. This relatively less deterioration was notably linked to the intact microstructure observed in the control samples of the Esma variety. Among the examined characteristics, the (6R) Tradition variety displayed larger cracks, accounting for approximately 6% of the seed volume, in contrast to 2% for the (2R) AC Metacalf variety. Furthermore, the gap between the endosperm and germ was more substantial in the hulled varieties (6R and 2R) of both classes at 11% and 10%, respectively. Consequently, the six-row hulled barley samples exhibited higher kernel air space. The volume of the husk and coat was found to be higher in the six-row hulled and hulless varieties (Tradition and AB Cattlelac) than in the two-row hulled and hulless (AC Metacalf and Esma) varieties.

Maximum and minimum air spaces or gaps were identified within the two row (2R) AC Metacalf and (6R) Tradition varieties both before storage and following an 8-week storage period. This observation highlights the variations in air space presence among the examined barley varieties. In the control samples, existing cracks were detected across all varieties, with the order of crack volume being as follows: (2R) AC Metacalf > (6R) Tradition > (6Rhl) AB Cattlelac > (2Rhl) Esma. These cracks appeared to originate from the crease region of the seeds. The emergence of cracks in this pattern may be attributed to external and internal stresses present within the seed structure due to the action of field operations, drying, or other unit operations [39]. These cracks were evident in the 3D segmentation visualization (dark blue). The progression of deterioration (red pixels) was observed to spread around these cracks and near the crease region, a trend that was similarly observed in 2D visualization. The 3D visualization of our data showed how existing irregularities like cracks in seeds widened due to the deterioration of endosperm at the end of storage period. In cereal food grains, the infection around the crease region may occur and this region was found vulnerable [40,41]. The germ of all varieties displayed signs of deterioration; a phenomenon demonstrated through 3D visualization. In these visualizations, the red pixels extended within and over the green pixels of germ. This deterioration within the germ could signify a decline in germination potential, ultimately impacting the malting value of the barley. Notably, the size of the germ differed between hulled and hulless varieties, with hulless varieties having larger germ sizes. Specifically, the germ size of Esma (2Rhl) was approximately 25% larger than that of AB Cattlelac (6Rhl). Although the germ volume remained relatively consistent after the 8-week storage period, slight deviations were observed in the germ volume of the hulled varieties in comparison to the control. This deviation could potentially be attributed to the presence of a larger deterioration volume within the segmentation results for these hulled varieties. It was also observed from Figure 5 that there were changes in seed volume at the end of the 8-week storage period, particularly with respect to spoilage in comparison to the control samples. Notably, the six-row varieties exhibited a greater change in volume (i.e., increased over control) compared to the two-row varieties. Air space was observed within the germ outer layer and germ in both the two-row and six-row hulled varieties over the germ. This could be helpful for germination but may make hulled seeds more susceptible to deterioration and ultimately might affect the seed viability. The results of the paired-t test indicated that there was a significant difference (*p* < 0.001) between the control and 8 wk samples for 6R and 2Rhl barley classes in the measured segmented seed volumes, but it was found non-significant for 6Rhl (*p* = 0.353) and 2R (*p* = 0.057). Similarly, the results of the paired-t test were carried out for microstructural feature cracks, and there were significant differences for the 6Rhl (*p* = 0.009) and 2R (0.043) barley classes but not for the 6R (*p* = 0.193) and 2Rhl (*p* = 0.153) barley classes. In the case of change due to spoilage, the significant differences were observed for 2R (*p* = 0.015), 2Rhl (*p* = 0.005) and 6Rhl (*p* = 0.004) but not for 6R (*p* = 0.055). It was observed from the statistical analysis that, in the case of spoilage, almost all the selected barely classes (four varieties) were found to be affected. Only barley class 2R, but not the other barley classes, showed a significant difference (*p* = 0.03) for husk content. Barely classes 2R (*p* = 0.043) and 6Rhl (*p* = 0.009) showed significant differences for the presence of cracks, but the other two classes (6R and 2Rhl) had no significant difference. The only barley class that showed a significant difference in endosperm was 2R (*p* = 0.034). But, in the case of germs and air spaces beneath the husk, all the barley classes showed nonsignificant differences between the control and 8 week samples.

To visually assess and quantify the differences between the control and 8-week stored samples, histograms were plotted for both categories (Figure 6). The data were deconvoluted in each histogram to locate seed features (endosperm, cracks, air, and spoilage) beneath the peaks to show significant changes and to support data analysis. This comparison aimed to examine the shift in peaks and their corresponding positions within the histograms. Notably, the shifting of peaks in the 8-week stored samples was particularly prominent, meaning the density was reduced and valuable insights were provided into the changes that had occurred due to deterioration [23]. Among the observed outcomes, the most significant shift in peaks was noted in the 8-week stored six-row and two-row hulled varieties. This observation served as validation, corroborating the previously noted changes evident in the X-ray images. The third peak of the histogram in the logscale histogram (Figure 6a) showed a change in height along with the shift. The lowering of the peak height can be attributed to a change in volume and density due to deterioration in storage. The pronounced shift in the peaks of these varieties further underscored the considerable impact of deterioration on the microstructure and composition of the barley samples during the storage period. The histogram analysis serves as a quantitative tool to elucidate changes in the distribution patterns and provides a direct visual representation of the effects of spoilage on the examined samples. The observed shifts in peaks substantiated the findings derived from the comprehensive analysis and supported the conclusions drawn regarding the influence of storage on the microstructural changes in the barley varieties. In the histograms of barley, the second peak corresponds to the seed endosperm, while the first peak represents the air spaces within the seed. Upon closer examination of all the histograms, a consistent pattern emerged: the grey value of 8-week stored samples decreased progressively. This decline in grey value signified a reduction in the density of the seed’s internal structure (Figure 6). The six-row varieties exhibited higher density when compared to their two row counterparts. This relationship between spoilage density alteration with time underscores the dynamic interplay between internal structural changes and the progression of spoilage within barley seeds.

### 3.2. Changes in Nutrient Distribution

The outcomes from synchrotron X-ray fluorescence imaging (XFI) are shown in Figure 7. The nutrient distribution map of one barley variety (6R) AB Cattlelac (control and 8-week stored samples) is shown as an example in Figure 7. It highlights the observed alterations in the distribution of nutrients, including K, Ca, Mn, Fe, Cu, and Zn within the 8-week stored barley when compared to the control. The methodology employed for the synchrotron XFI results has been verified to yield statistically consistent findings when compared to the analytical methods [42]. The colour scales were used for visual representations, which enabled clear visualization of the variations in nutrient distribution across the barley samples. The analyzed images effectively illustrated how the distribution of nutrients within the barley seeds changed due to deterioration. By capturing and presenting these variations, the synchrotron XFI method enhanced our understanding of the impact of spoilage on the nutrient distribution within barley seeds. This information can have broader implications for assessing the nutritional quality of stored barley and making informed decisions regarding post-harvest management strategies.

The nutrient K was observed to be most abundant in all control samples of the selected barley varieties, as depicted in Figure 7. It displayed a well-distributed presence across all seed components, namely the endosperm, seed coat, and embryo. The distribution sequence (high to low) as per intensity was (6R) AB Cattlelac > (6Rhl) Tradition > (2Rhl) AC Metacalf > (2R) Esma. Previous research has indicated that K tends to concentrate in the scutellum, seed coat, and vascular bundle within cereal grains [43]. However, K was found at lower intensities in the husk of hulled varieties. Our study suggests that the distribution of K was influenced by spoilage. Specifically, in the case of variety AC Metacalf, the impact on K distribution was relatively minor compared to other varieties, as K distribution was found visible in both the seed coat and endosperm regions. In the hulled varieties, the distribution of nutrient K in the husk remained unchanged. Interestingly, the distribution of K within the endosperm was reduced near or in the region of the endosperm, as depicted in Figure 7. This suggests that spoilage-induced changes due to moisture resulted in damage to the endosperm region (which was observed earlier in SR-µCT), leading to alterations in K distribution. In the hulless varieties, K was found to be present in the seed coat after 8 weeks of storage. These distribution patterns align with the findings from previous studies [34,44,45].

The iron (Fe) concentrations exhibited distinct intensities in the seed coats (aleurone) of all samples. The control barley samples, i.e., those taken prior to storage, showcased greater-intensity Fe distributions within the seed coat in comparison to the 8-week stored samples. In the latter scenario, there was a noticeable reduction in the intensity of Fe. This phenomenon can be further substantiated by considering the example of the six-row barley variety, as depicted in Figure 7. Fe was not detected in the husk region of the hulled varieties belonging to both the two-row and six-row classes. The distribution of Fe abundance based on intensity was in the following order: (6Rhl) Tradition > (2Rhl) AC Metacalf > (6R) AB Cattlelac > (2R) Esma. Previous research has reported that Fe is primarily concentrated in the aleurone layer [43,46]. Our study aligns with these findings, as we observed similar results in our analysis, but this holds true for only the six-row varieties in the study. The two-row barley varieties, both hulled and hulless, exhibited the presence of Fe in the endosperm at lower intensities compared to in the aleurone region. Notably, these varieties displayed a higher disparity in Fe distribution between the 8-week stored and control samples.

Subsequently, the essential nutrients Mn and Zn were visualized across thin sections of the barley sample. Within the control samples, Zn was notably concentrated in the aleurone layer and embryo region more than in the endosperm. In contrast, Mn exhibited a more uniform distribution across all sections of the control samples. It was found that, in the control samples, the distribution of Zn was in the following order (most abundant to least abundant): (6R) AB Cattlelac > (2Rhl) AC Metacalf > (2R) Esma > (6Rhl) Tradition. In contrast, the distribution of Mn was in the following order: (2Rhl) AC Metacalf > (6R) AB Cattlelac > (6Rhl) Tradition > (2R) Esma. However, an evident degradation of both Zn and Mn was observed in the 8-week stored samples of the four barley varieties. Comparatively, the degradation of Mn was more pronounced than that of Zn in the stored samples. Among the varieties, the AC Metacalf variety exhibited the highest degree of degradation, particularly in the case of Zn, while the Esma variety showed the least degradation of Zn. The images revealed that Zn was also localized in the embryo and crease regions of the barley seeds. These observations align with those of earlier studies that reported similar distribution patterns of Zn and Mn in barley samples [34,43,45]. Notably, all the stored samples exhibited a lower abundance of Mn compared to Zn at the end of 8-week storage. The AC Metcalf variety exhibited more substantial variations, indicating changes in the distribution of Zn and Mn in comparison to the control samples, as compared to the other three varieties.

In the results, the degradation of micronutrients was observed due to spoilage with increasing storage period. Micronutrients are considered essential for plant defense to diseases, which have been previously documented for Mn, Cu, Fe, and Zn [47,48,49]. Mn was found responsible in the production of phenolic compounds which assist in plant defense mechanisms [50] and Zn has been reported to play a major role in plant immune responses [51]. Both Zn and Mn were found concentrated in the crease region of the four barley control samples but, later after 8 week of storage, their abundance decreased, which might be due to spoilage starting near the crease region of the grain. The crease features of grains have a great impact on their post-harvest quality [40]. In hulless varieties, a large gradient was observed between the control and 8-week stored barley compared to the hulled varieties. Therefore, hulless varieties under both classes of barley may perform comparatively weaker in storage and be more susceptible to spoilage than the hulled varieties, and similar results were found when hulled and hulless barley were stored for 12 month [13]. The variations that were observed in nutrient distribution due to deterioration could be linked to fungal infection. One of the reasons for variation could be due to the growth of fungus on the barely seeds, which per the literature, might deprive plant cells of iron and other essential nutrients that support their metabolic activities [52]. In our study, the structural changes were found to be maximum in hulled varieties compared to the hulless varieties, and this could be due to the prior condition of the barley grains, as discussed earlier. In all hulled and hulless varieties, changes, or reductions in the distribution of other nutrients (Ca, K, Fe, and Cu) were observed in the spoiled samples. These vital nutrients are responsible for healthy grain and the growth of barley [53]; hence, their degradation in storage means a loss of germination, and a loss in germination of malting barley below 95% is not recommended if it is used for malt production [15]. The utilization of synchrotron X-ray fluorescence imaging (SR-XFI) proved valuable in accurately mapping the changes occurring during post-harvest storage in barley due to spoilage.

### 3.3. Biochemical Changes

The outcomes of the mid-IR spectroscopy analysis are shown in Figure 8, Figure 9 and Figure 10. These figures illustrate the changes in protein, carbohydrates, and lipids in 8-week stored barley samples, as compared to their corresponding control samples. The analysis is based on the collected spectra, and the focus lies in evaluating alterations in these key biochemical components due to spoilage at the end of 8 weeks of storage. The spectral region ranging from 1800 to 900 cm^−1^ was used in the analysis, as depicted in Figure 8. This IR region is called the fingerprint region and its range definition can vary from between 1800–1500 cm^−1^ and 800–600 cm^−1^. This region was selected due to its relevance, featuring absorption bands dominated by contributions from the protein, lipid, and carbohydrate contents [54]. Lipids, proteins, and carbohydrates are marked by distinct absorption peaks at 1740 cm^−1^, 1651 cm^−1^, and 1080-1023 cm^−1^ in the selected barley variety control and 8-week stored samples [55]. These spectral analyses provide a means to track and quantify the changes in the key biochemical constituents over the 8-week storage duration. The shifts in absorption bands can offer valuable insights into the alterations occurring in the barley samples composition, thereby aiding in a comprehensive understanding of the effects of storage on their nutritional and biochemical characteristics.

The peaks at 1158, 1081, and 1024 cm^−1^ represent the carbohydrate region. The bands located at 1081 and 1024 cm^−1^ are attributable to starch components, while the band at 1150 cm^−1^ is linked to the stretching vibrations of single C-C, C-O-H, and C-O-C bonds [37]. The control samples exhibited a distinct amount for carbohydrates, which was in the following order (most to least): (2Rhl) Esma > (2R) AC Metacalf > (6Rhl) AB Cattlelac > (6R) Tradition. This means that, before storage, the two-row barley varieties had higher carbohydrates compared to the six-row varieties. However, in the case of the 8-week stored samples, a notable decrease in absorption at 1080 cm^−1^ for carbohydrates was observed and the order changed to the following: (2R-8wk) AC Metacalf > (6Rhl-8wk) AB Cattlelac > (2Rhl-8wk) Esma > (6R-8wk) Tradition. This change in absorption indicated shifts in carbohydrate content due to spoilage after 8-week storage. The spoiled cereals showed lower absorbance for biochemical components (carbohydrates, protein, and lipids), as reported in an earlier study [56]. The protein spectra in the regions of 1600–1500 cm^−1^ and 1700–1600 cm^−1^ were investigated, with amide I at 1650 cm^−1^ and amide II at 1540 cm^−1^ [55]. To evaluate the protein structure, second derivatives of the spectra were plotted at 1700-1600 cm^−1^, as illustrated in Figure 9. The β-sheets and α-helical secondary protein structures exhibited peaks at 1636 cm^−1^ and 1656 cm^−1^, respectively. The selected barley varieties demonstrated almost similar patterns of secondary protein structures, except for the 8-week stored (6R) Tradition and (2Rhl) AC Metacalf barley varieties. There was a considerable variation observed in the normalized absorbance of 8-week stored barley and the control in the second derivative spectra, which can be inferred from individual spectra, as shown in Figure 9A–D. The hulled barley class (6R and 2R) showed lower absorbance for the β-sheets and α-helical secondary protein structures (Figure 9A,C). This observation signified changes in the protein secondary structures due to spoilage. The normalized absorbance displayed considerable variation in both the spoiled samples and the control samples after 8 weeks of storage, as seen in Figure 8. In contrast, the control samples exhibited a consistent trend without major variations in peaks, unlike the altered pattern observed in the stored samples, as shown in Figure 8. This comparison highlights the impact of storage-induced spoilage on the normalized absorbance and spectral characteristics of carbohydrates and proteins in barley varieties.

A distinct pattern emerged in terms of lipid absorption at 1740 cm^−1^ for the selected barley varieties. The lipid peak at 1740 cm^−1^ corresponds to the ester C = O stretching in triglycerides for all polyhydroxy aldehydes [57,58]. This divergence in absorption patterns can be attributed to the unique composition and structure of the hulled and hulless barley varieties, influencing the spectral response during spoilage. The lowest absorption (1740 cm^−1^) for lipids was observed for the 8-week stored (6Rhl) AB Cattlelac variety and the highest absorption was in the control (6R) Tradition variety followed by the 8-week stored (6Rhl) Tradition variety. Previous research findings [54,56] align with the present study’s observation of lower absorbance for key biochemical components (carbohydrates, protein, and lipids) in spoiled cereals. This shift in peaks during spoilage may arise from changes in the metabolic activity, as well as variable defense mechanisms inherent to each barley variety [29]. In the deterioration process, fungi can absorb and metabolize a diverse range of soluble carbohydrates and insoluble ones like starches and cellulose [59]. It was also reported that fungal spoilage of cereals can degrade protein and carbohydrates by 3–19% and 77% of their original value [60].

A Principal Component Analysis (PCA) was conducted, and the outcomes are depicted in Figure 10. This figure illustrates the comparison between the PC1 and PC2 scores of the control and 8-week stored barley samples. The PCA is a widely employed technique in IR spectroscopy to visualize the distribution of data points [29,56]. In this context, PCA serves as a valuable tool to analyze and comprehend the patterns and relationships within the collected spectral data. The principal components PC1, PC2, and PC3 accounted for approximately 52%, 14%, and 7% of the explained variance, respectively. Almost all the stored samples were inclined towards the right side as compared to the control. These results are in line with previously reported findings in the context of cereal analyses [29,56]. For the Esma and Tradition varieties, the PCA scores of the stored samples were situated at the far right; these findings were in line with the results of the SR-µCT data, where large changes due to deterioration in the seed microstructure were observed. From the data loading of PC1, PC2, and PC3 shown in Figure 10, notable observations can be made in the carbohydrate (1200–900 cm^−1^) and protein (1700–1500 cm^−1^) regions. It was used in the differentiation of stored samples and control samples based on biochemical changes. The application of PCA, in conjunction with the identified mid-IR peaks, presents a potential avenue for the characterization of stored grains. By leveraging the observations derived from this study, PCA could prove to be a valuable technique for distinguishing and categorizing different types of spoilage, particularly those associated with fungal infection, based on mid-IR spectroscopy data. This approach offers a promising means to enhance the understanding of spoilage processes and to facilitate the development of strategies for identifying and addressing issues related to food grain quality and safety.

## 4. Conclusions

This study’s findings underscore the crucial significance of assessing the initial post-harvest quality of barley prior to its storage. While the adverse impact of unfavorable moisture conditions on barley storage is widely acknowledged, our study stands out for providing a comprehensive mapping of actual deterioration and the changes in barley microstructure through synchrotron X-ray techniques. This knowledge holds substantial promise in guiding the formulation and execution of storage strategies. The atlas of high-resolution images developed in our study, encompassing the most prevalent malt and feed barley classes, serves as a foundational framework for the planning of post-harvest processing procedures for barley. A notable conclusion is that the presence of a hull cannot guarantee the protection of barley seeds if their structural integrity is already compromised. Moreover, our results emphasize that some hulless varieties may perform better in storage conditions. In our research, we successfully established a connection between microstructural changes and the corresponding nutritional and biochemical alterations resulting from spoilage over a 8-week storage period. The hulled barley varieties showed more deterioration in microstructure than the hulless varieties of barley, where a direct correlation between microstructural changes and alterations in nutritional content was found. All selected barley classes showed changes in distribution of nutrients (Ca, Fe, K, Mn, Cu, and Zn), but the two-row AC Metcalf variety exhibited more substantial variations in the nutrient distribution (Zn and Mn) than the other three varieties during storage. The two-row class barley varieties showed more changes in biochemical components (protein, lipids, and carbohydrates) than the six-row class varieties. These findings have practical implications for optimizing post-harvest storage practices in the barley industry, facilitating enhanced quality preservation and management. The future scope of our work is to train synchrotron data for the development of machine learning algorithms for artificial intelligence (AI) segmentation of spoilage and the identification of heathy barely for grain storage and malting industry.

## Data Availability

High-resolution imaging data are available upon request to the corresponding author.
